# Methanolic Extract of the Nutritional Plant (*Diospyros kaki* Thunb.) Exhibits Anticancer Activity by Inducing Mitochondrial Dysfunction in Colorectal Cancer Cells

**DOI:** 10.3390/nu16213742

**Published:** 2024-10-31

**Authors:** Stefano Bianchini, Federica Bovio, Stefano Negri, Leonardo Bisson, Anna Lisa Piccinelli, Luca Rastrelli, Matilde Forcella, Paola Fusi

**Affiliations:** 1 Department of Biotechnology and Biosciences, University of Milano-Bicocca, Piazza della Scienza 2, 20126 Milano, Italy; stefano.bianchini@unimib.it (S.B.); federica.bovio@unimib.it (F.B.); 2 National Biodiversity Future Center (NBFC), 90133 Palermo, Italy; stefano.negri@univr.it (S.N.); leonardo.bisson@univr.it (L.B.); apiccinelli@unisa.it (A.L.P.); rastrelli@unisa.it (L.R.); 3 Department of Biotechnology, University of Verona, Strada Le Grazie 15, 37134 Verona, Italy; 4 Department of Pharmacy, University of Salerno, Via Giovanni Paolo II, 132, Fisciano, 84084 Salerno, Italy; 5 Integrated Models for Prevention and Protection in Environmental and Occupational Health (MISTRAL), Interuniversity Research Center, 25121 Brescia, Italy

**Keywords:** colorectal cancer, oxidative stress, mitochondria, apoptosis, flavonoids, quercetin, kaempferol

## Abstract

**Background/Objectives:** *Diospyros kaki*, the most widely cultivated species of persimmon, has been long used in traditional medicine since its leaves’ extracts contain high amounts of flavonoids and terpenoids, endowed with several beneficial effects. However, its anticancer activity towards colorectal cancer (CRC) has not been investigated in depth. **Methods:** The effect of a methanolic extract of *D. kaki* leaves, rich in kaempferol and quercetin derivatives, have been evaluated on an E705 CRC cell line, representative of most CRC patients, and on SW480 cells, carrying a KRAS-activating mutation. **Results:** This extract is effective in reducing tumor cells’ viability without affecting the healthy mucosa cell line CCD 841. In fact, Western blot experiments showed its ability to induce apoptosis in cancer cells by increasing oxidative stress and disrupting mitochondrial functionality, as shown by reactive oxygen species measurement and Seahorse analysis. **Conclusions:** With the aim of increasing healthspan, as well as the substantial societal and macroeconomic costs associated with cancer, our results could pave the way to a role for *D. kaki* extract in both CRC treatment and prevention.

## 1. Introduction

One of the major societal, public health, and economic problems of the 21st century is cancer, with 20 million cases estimated in 2022 and a prediction of over 35 million new cases by 2050 [1]. Colorectal cancer (CRC), with more than 1.9 million new cases in 2022, is the third most common cancer worldwide and the second deadliest [2]. Over 80% patients are affected by sporadic CRC, whose incidence is increasing in patients under 50 years of age, with enhanced mortality compared to older patients [3]. Epidemiological studies show a higher incidence in Western countries and a steadily rising incidence in countries undergoing major transition, indicating the strong role of environmental factors [1,2]. Moreover, recent data strongly suggest that lifestyle plays a very important role in CRC pathogenesis. In fact, excessive red meat, alcohol intake and smoking, as well as being overweight and a sedentary lifestyle promote CRC [2]. Conversely, regular physical activity, a diet rich in fruits, vegetables, fiber and fish, as well as adequate vitamin supply, have been found to be protective towards CRC [4,5].

The current CRC treatment requires a multimodal approach with surgery usually being the primary step; this is frequently combined with adjuvant therapy based on cytotoxic chemotherapy drugs, like oxaliplatin, or targeted therapies, such as anti-EGFR monoclonal antibodies [6]. However, these drugs are associated with serious side effects and chemoresistance. Moreover, anti-EGFR monoclonal antibodies show no efficacy towards patients carrying *RAS*/*BRAF* mutations, whose prognosis is generally more unfavorable [7]. For these reasons, the search for new and more effective therapies has turned in recent years to plant extracts, a very interesting source of phytochemicals which could reduce conventional drugs’ dosages and toxicity [8].

Oriental persimmon, *Diospyros kaki* Thunb. (Ebenaceae), is the most widely cultivated species of its genus. Its leaves have long been used in traditional medicine to treat infectious diseases, bites, constipation, hemorrhages and strokes [9]. Moreover, in Asian cultures, the leaves’ extracts are also used to make tea and as food additives [10]. They contain high amounts of flavonoids and terpenoids, endowed with potential antioxidant, antihypertensive, anti-inflammatory, anticancer, antidiabetic, antiallergic and antimicrobial effects [9]. More recently, many in vitro and in vivo studies have assessed different potential health benefits of persimmon leaves [9].

As regards anticancer activity, ethanolic extract of *D. kaki* L. leaves has been shown to induce apoptosis in prostate cancer PC-3 cells by increasing oxidative stress [11] and to activate c-Jun *N*-terminal kinase (JNK) [12]. These effects have been attributed to flavonoids, the main components of persimmon leaf extracts. However, polysaccharides have also shown an anticancer effect, suppressing Transforming Growth Factor-beta 1 (TGF-β1)-induced epithelial-to-mesenchymal transition in A549 lung cancer cells [13]. Other studies have shown that persimmon leaf ethanolic extracts are endowed with antioxidant activity and free-radical scavenging ability [14]. Moreover, ethanol extracts of *D. kaki* leaves have been shown to inhibit epithelial-to-mesenchymal transition in hepatocellular carcinoma cell cultures [15].

The anticancer activity of *D. kaki* leaf extracts towards colorectal cancer (CRC) has not been investigated in depth. A preventive effect towards CRC was suggested by Direito and collaborators, who detected anti-inflammatory properties in persimmon phenolic extracts, as well as the ability to impair cell proliferation and invasion in colon carcinoma HT-29 cells [16]. Chen and coworkers have reported that flavonoids obtained from persimmon leaves can induce in HCT-116 CRC cells apoptosis by increasing intracellular ROS, causing damage to the cell membrane and rupture of the nuclear membrane [10]. Keskin and collaborators have shown that silver nanoparticles coated with persimmon leaf extracts reduce Caco2 cells viability in a dose dependent way [17]. Moreover, Park and coworkers showed that *D. kaki* calyx, a plant byproduct containing high polyphenol levels, suppressed the proliferation of different human CRC cell lines, decreasing cyclin D1 expression through Wnt signaling [18].

In this work, we show that persimmon leaf extract is effective in reducing viability of two different CRC cell lines: E705 cells, representative of most CRC patients being *KRAS*, *NRAS*, or *BRAF* wild type but carrying a silent mutation in the *PIK3CA* gene, and SW480 cells, carrying a *KRAS* activating mutation which normally leads to a less common but more aggressive form of CRC. Persimmon leaf extract triggers apoptosis in both cell lines by increasing oxidative stress and disrupting mitochondrial functionality.

## 2. Materials and Methods

### 2.1. Cell Cultures

The human colorectal cancer cell lines E705 (kindly provided by Fondazione IRCCS Istituto Nazionale dei Tumori, Milan, Italy) and SW480 (CCL-228^™^ ATCC, Manassas, VA, USA) were grown in RPMI 1640 medium supplemented with heat-inactivated 10% FBS, 2 mM L-glutamine, 100 U/mL penicillin, and 100 µg/mL streptomycin. The human healthy colon mucosa cell line CCD 841 (CRL-1790^™^ ATCC, Manassas, VA, USA) was grown in EMEM medium supplemented with heat-inactivated 10% fetal bovine serum (FBS), 2 mM L-glutamine, 0.1 mM non-essential amino acids, 100 U/mL penicillin, and 100 µg/mL streptomycin. All cell lines were maintained at 37 °C in a humidified 5% CO_2_ incubator. Cell lines were validated by short tandem repeat profiles that are generated by simultaneous amplification of multiple short tandem repeat loci and amelogenin (for gender identification). All the reagents for cell cultures were supplied by EuroClone (EuroClone S.p.A, Milan, Italy).

### 2.2. Plant Material and Extract Preparation

Leaves of *D. kaki* Thunb. were collected from a fruit-bearing tree growing in the botanical garden of Padua (via Orto botanico 15, 35123 Padua, Italy) and immediately frozen in dry ice. The plant material was ground in liquid nitrogen through an A11 basic analytical mill (IKA-Werke, Staufen, Germany) and 1× *g* of the resulting frozen powder was extracted with 10 mL of LC-MS grade methanol (Honeywell, Seelze, Germany). The sample was vortexed for 30 s, sonicated for 10 min at 40 kHz in an ultrasonic bath (SOLTEC, Milano, Italy) with ice and centrifuged at 4 °C for 10 min at 14,000× *g*. The supernatant was split into 1 mL-aliquots, each deriv from 100 mg of fresh leaves, and dried using a speed-vac system (Heto-Holten; Hillerød, Denmark).

### 2.3. UHPLC-DAD-HRMS/MS Analysis

Untargeted analysis of *D. kaki* extract was performed on a Vanquish Flex UHPLC system interfaced to Diode Array Detector FG and Orbitap Exploris 120 mass spectrometer (ThermoFisher Scientific, Milano, Italy) equipped with a heated electrospray ionization source (HESI-II). A Kinetex C18 column (2.1 × 100 mm, 2.6 μm; Phenomenex, Bologna, Italy), protected by a C18 Guard Cartridge (2.1 mm I.D.) and thermostated at 30 °C, and a binary gradient (0–3 min, 2% B; 3–5 min, 2–13% B; 5–9 min, 13% B; 9–13 min, 13–18% B; 13–17 min, 18–30% B; 17–20 min, 30% B; 20–30 min, 30–40% B; 30–38 min, 40–60% B; 38–40 min, 68–98% B flow rate of 500 µL min^−1^ and injection volume of 5 µL) of H_2_O (A) and MeCN (B), both containing 0.1% of HCOOH, were employed for the chromatographic separation.

The mass spectrometer was operated in positive and negative ionization modes using a Full MS data-dependent MS/MS acquisition mode with a stepped collision energy HCD (20, 40 and 60). The resolution of the Full MS scans (scan range 150–1500 *m*/*z*) and dd-MS2 scans was set at 30k (FWHM). Instrument control and spectra acquisition were carried out using Xcalibur software (Version 4.4, ThermoFisher Scientific, Waltham, MA, USA). UV spectra were acquired in the 200–600 nm range.

Detected compounds were characterized based on HRMS data (accurate masses, probable molecular formulas and product ions) and retention times. The identification level was established following the metabolomics standards initiative (MSI): level 1, unambiguous identification with reference standards; level 2, tentative identification by comparing MS2 data with literature or spectral databases; level 3, tentative identification by spectral similarity to chemical class of compounds and chemotaxonomic data.

### 2.4. Viability Assay

The different cell lines were seeded in 96-well microtiter plates at a density of 1 × 10^4^ cells/well, cultured in complete medium and the following day treated for 24 h with *D. kaki* extract, solubilized in pure ethanol, at a concentration ranging between 0 and 400 μg/mL. Ethanol concentration in the wells was 0.5% in both treated and untreated cells. Cell viability was investigated using an MTT (3-(4,5-Dimethylthiazol-2-yl)-2,5-Diphenyltetrazolium Bromide) in vitro toxicology assay kit (Merck KGaA, Darmstadt, Germany) according to the manufacturer’s protocols. Absorbance was measured at 570 nm using a Spectrostar Nano Microplate Reader (BMG LABTECH, Ortenberg, Germany) after a 4 h incubation for CCD 841 and 2 h for E705 and SW480 cell lines, upon formazan crystal solubilization. Cell viability was expressed as a percentage against untreated cells used as control. For each cell line, the experiments were performed in three technical repeats per treatment for each biological replicate and at least three independent biological replicates were carried out.

### 2.5. SDS-PAGE and Western Blotting

For Western blot analysis, SW480 and E705 cells were seeded at a density of 6 × 10^5^ cells/60 mm dish and treated with 200 and 400 μg/mL *D. kaki* extract 24 h after seeding. At the end of the 24 h treatment, cells were rinsed with ice-cold PBS (10 mM K2HPO4, 150 mM NaCl, pH 7.2) and lysed on ice in RIPA buffer (50 mM Tris-HCl pH 7.5, 150 mM NaCl, 1% NP-40, 0.5% sodium deoxycholate and 0.1% SDS) containing 1 μM leupeptin, 2 μg/mL aprotinin, 1 μg/mL pepstatin, 1 mM PMSF and a phosphatase inhibitor cocktail (Merck KgaA, Darmstadt, Germany). Subsequently, homogenates were obtained by passing the solution 5 times through a blunt 20-gauge needle fitted to a syringe and then centrifuging it at 15,000× *g* for 30 min. Supernatants were analyzed for protein content by the BCA protein assay [19]. SDS-PAGE and Western blotting were carried out by standard procedures [20]. The following primary antibodies, anti-Bcl-2 (#15071), anti-caspase-3 (#14220), anti-*P*-ERK (#4370) and anti-ERK (#4695), were purchased by Cell Signaling Technology, Danvers, MA, USA and used at a dilution of 1:1000, while anti-vinculin (V9131), purchased by Merck KgaA, Darmstadt, Germany was used at a dilution of 1:5000. Secondary antibodies IgG HRP anti-rabbit (#7074) and IgG HRP anti-mouse (#7076), purchased by Cell Signaling Technology, Danvers, MA, USA, were used at a dilution of 1:8000. Protein levels were visualized using an ECL detection system (EuroClone S.p.A, Milan, Italy) and quantified by densitometry of immunoblots using ImageStudio^™^ software version number 5.5 (LI-COR Biosciences, Lincoln, NE, USA).

### 2.6. Intracellular Reactive Oxygen Species (ROS) Measurement

Dichlorofluorescein diacetate (H_2_DCFDA) dye has been used for total intracellular reactive oxygen species (ROS) detection. SW480 and E705 cell lines were seeded in 96-well black microplates with clear bottoms at a density of 1 × 10^4^ cells and 2 × 10^4^ cells per well, respectively. After twenty-four hours following seeding, cells were incubated with 5 µM H_2_DCFDA in PBS for 30 min in the dark at 37 °C; then, they were rinsed in PBS and treated for 4 h with 200 and 400 μg/mL *D. kaki* extract before fluorescence (λem = 485 nm/λex = 535 nm) was measured using a fluorescence microtiter plate reader (VICTOR X3, PerkinElmer, Akron, OH, USA). Normalization was performed on total protein content, measured with a Bradford assay [21]. For each cell line, the experiments were performed in three technical repeats per treatment for biological replicate and at least three independent biological replicates were carried out. All chemicals were supplied by Merck KGaA, Darmstadt, Germany.

### 2.7. Glutathione Detection

Total glutathione, oxidized glutathione (GSSG) and reduced glutathione (GSH) content was measured on colorectal cancer cells treated with 200 and 400 μg/mL *D. kaki* extract for 24 h. In detail, SW480 and E705 cells were seeded in 6-well plates at a density of 2 × 10^5^ cells/well and, the day after seeding, treated with *D. kaki* extract for 24 h. At the end of the treatment, cells were harvested by trypsinization, washed with PBS and then glutathione measurements were performed as described in Bovio et al., 2024 [22]. Three technical replicates were performed for each biological replicate. All chemicals were supplied by Merck KGaA, Darmstadt, Germany.

### 2.8. Mitochondrial Transmembrane Potential (MTP) Evaluation

SW480 and E705 cells were plated at a density of 1 × 10^4^ cells per well in 96-well black microplates with clear bottom and 24 h later treated with *D. kaki* extract at a final concentration of 200 and 400 μg/mL for a 24 h treatment. Then, treated cells were incubated for 20 min at 37 °C and 5% CO_2_ in the dark with 40 nM 3,3′-dihexyloxacarbocyanine iodide (DiOC6), a potential sensitive carbocyanine that accumulates in mitochondria due to their negative membrane potential [23,24]. Plates were rinsed in PBS twice and fluorescence was measured at emission 485 nm and excitation 535 nm in end point mode using a fluorescence microtiter plate reader (VICTOR X3, PerkinElmer, Akron, OH, USA). Normalization was performed on total protein content, measured with a Bradford assay [21], and three technical repeats were carried out for each biological replicate. All chemicals were supplied by Merck KGaA, Darmstadt, Germany.

### 2.9. Seahorse Mito Stress Test and ATP Rate Assay

For the evaluation of mitochondrial parameters as well as the total ATP production, distinguishing between the amount derived from oxidative phosphorylation and glycolysis, Agilent Seahorse XF Cell Mito Stress Test Kit and XF ATP Rate Assay Kit were performed according to manufacturer protocols.

In brief, SW480 and E705 cells were seeded in Agilent Seahorse 96-well XF cell culture microplates at a density of 2 × 10^4^ cells/well in 180 µL of growth medium, allowed to adhere for 24 h in a 37 °C humidified incubator with 5% CO_2_ and treated with 200 and 400 μg/mL *D. kaki* extract for a further 24 h. Before running the assay, the Seahorse XF Sensor Cartridge was hydrated and calibrated with 200 µL of Seahorse XF Calibrant Solution in a non-CO_2_ 37 °C incubator. Moreover, at the end of the treatment, the medium was replaced with 180 μL/well of Seahorse XF RPMI Medium with a pH of 7.4 containing 1 mM pyruvate, 2 mM L-glutamine and 10 mM glucose, and the Seahorse analyses were carried out.

For each biological replicate, a technical quadruplicate was performed and data were normalized on total protein content, quantified by a Bradford assay [21]. All the kits and reagents were purchased by Agilent Technologies, Santa Clara, CA, USA.

### 2.10. Statistical Analysis

The experiments were carried out in biological triplicate and the samples were compared to their reference controls. Data were tested using a one-way ANOVA followed by Dunnett’s multiple comparison procedure (GraphPad Prism Software v. 8.0.2) and results were considered statistically significant at *p* < 0.05.

## 3. Results and Discussion

### 3.1. Profiling of Bioactive Compounds of D. kaki Leaf Extract

**Figure 1:**
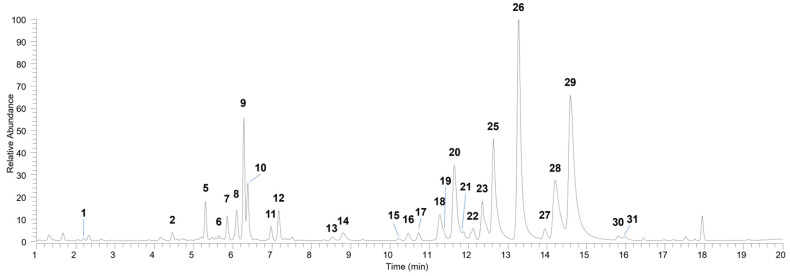
UHPLC-(―)-HRMS profiles of *Diospyros kaki* extract (full range MS, *m*/*z* 150–1500).

The profile of specialized metabolites in *D. kaki* leaf extract was defined using untargeted UHPLC-HRMS/MS analysis. A total of thirty-one compounds were tentatively identified based on HRMS/MS data and comparison with literature or database data, chemo-taxonomic data and the use of available standard compounds. The main compounds are listed in Table 1, and the UHPLC- HRMS profile is shown in Figure 1.

Flavonol glycosides (13–15, 18, 19–31), mainly consisting of kaempferol, quercetin, myricetin and laricitrin derivatives, were found to be the most abundant and representative compounds. They exhibited diagnostic product ions in the (−)-HRMS^2^ spectra resulting from the retro Diels-Alder reaction (^1,3^A^−^, ^1,2^A^−^ and ^1,2^B^−^) of the flavonol skeleton, as well as from the loss of the sugar moieties [25]. Kaempferol derivatives such as astragalin (26), one of its isomers (25) and two galloyl derivatives (28 and 29) were the major flavonol glycosides in *D. kaki* leaf.

Flavan 3-ols, including gallocatechin (2), catechin (7) and four B-type dimers (1, 3, 4 and 6), were also identified in the extract based on their characteristic fragmentation pattern [26].

The results of the untargeted analysis conducted in this study reveal a profile consistent with previously reported data on the composition of *D. kaki* leaves [9,27].

### 3.2. D. kaki Extract Reduces CRC Cells Viability, Triggering Apoptosis

**Figure 2:**
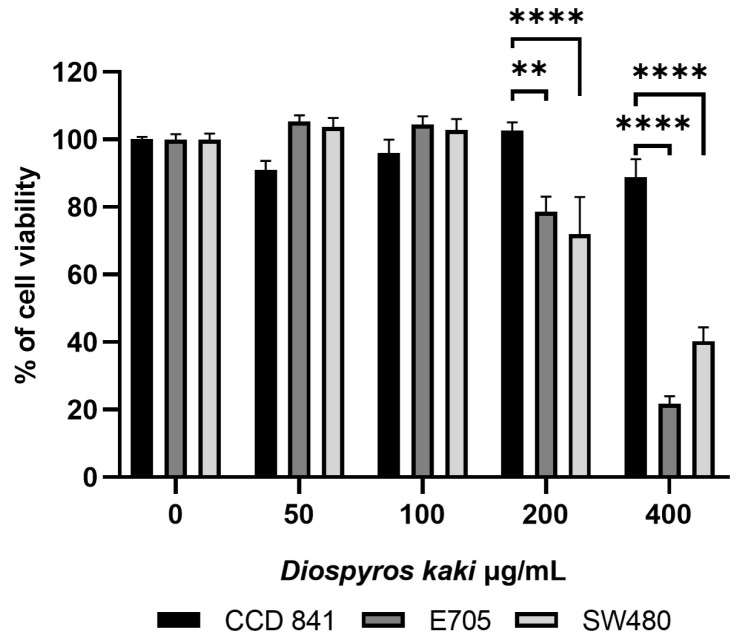
Cell viability of the healthy colon mucosa CCD 841 cell line and the colorectal cancer E705 and SW480 cell lines treated with *D. kaki* extract (0–400 µg/mL) for 24 h. Average values ± SE from three biological replicates are shown and three technical replicates are included in each biological replicate. Statistical significance: ** *p* < 0.01, **** *p* < 0.0001.

As reported in Figure 2, MTT tests performed on E705 and SW480 cells treated with different D. kaki concentrations showed a reduction in cell viability, starting from 200 µg/mL extract. Following treatment with 400 µg/mL extract, a reduction of over 70% and 60% was achieved for E705 and SW480 cells, respectively. Viability of healthy colon mucosa CCD 841 cells remained around 90% even at the highest extract concentration. This selectivity towards cancer cells is in accordance with previous works [10] and could explain the few side effects found in tumor-bearing mice treated with a flavonoid-enriched D. kaki extract compared to the severe side effects of cyclophosphamide [28].

**Figure 3:**
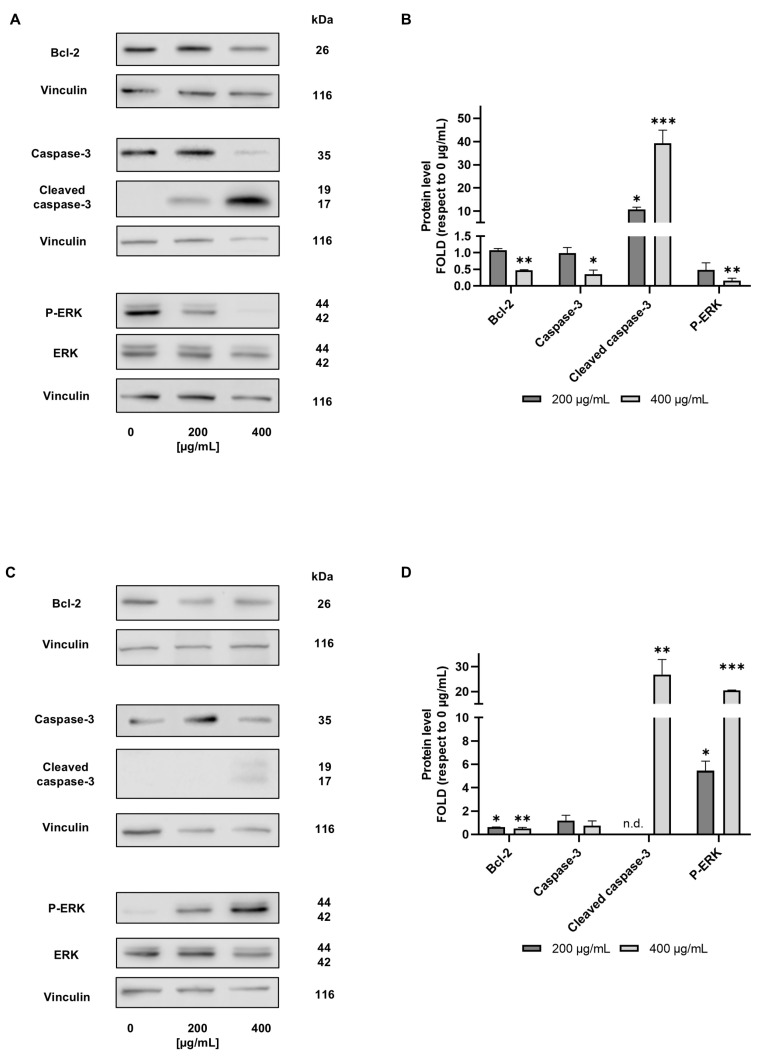
Representative Western blot analysis performed on E705 (**A**) and SW480 (**C**) cell lines untreated and treated for 24 h with 200 and 400 µg/mL of *D. kaki* extract. Protein extracts were separated on 12% acrylamide/bis-acrylamide SDS-PAGE and the nitrocellulose membranes were probed with anti-Bcl-2, anti-caspase-3, anti-*P*-ERK and anti-ERK antibodies. Vinculin was used as a loading control. Quantifications of the immunoblots are represented as fold respect to the untreated condition. Data are shown as mean ± SE from three biological replicates (**B**,**D**), except for Bcl-2 in the E705 line (two replicates). n.d.: not detected. Statistical significance: * *p* < 0.05, ** *p* < 0.01, *** *p* < 0.001.

Western blotting, reported in Figure 3, showed for both cancer cell lines the downregulation of Bcl-2 and a decrease in the level of full-length caspase-3, following its proapoptotic cleavage. Cleaved caspase-3 was found to increase 40-fold and 25-fold in E705 and SW480 cells, respectively, thus demonstrating the proapoptotic effect of D. kaki extract. These data are in accordance with a previous work on prostate cancer PC-3 cells, reporting the increase in cytochrome c release, which suggests the activation of the mitochondrial-dependent apoptotic pathway [11]. However, while in E705 cells, treatment with D. kaki extract decreased ERK phosphorylation, suggesting the downregulation of the EGFR downstream pathway, in SW480 cells, ERK phosphorylation was found to increase. A role for ERK activation in promoting apoptosis has been previously proposed and many compounds from plant extracts, including betulinic acid, quercetin, kaempferol and piperlongumine, have been reported to promote apoptosis through ERK activation [29]. Moreover, in a previous work by our group, we observed a particularly marked ERK activation triggering apoptosis in SW480 cells treated with polyphenol-enriched fractions of extracts of Cinnamomum cassia bark, Cinnamomum zeylanicum bark and Cinnamomum cassia buds [30].

### 3.3. D. kaki Extract Increases Oxidative Stress in CRC Cells

**Figure 4:**
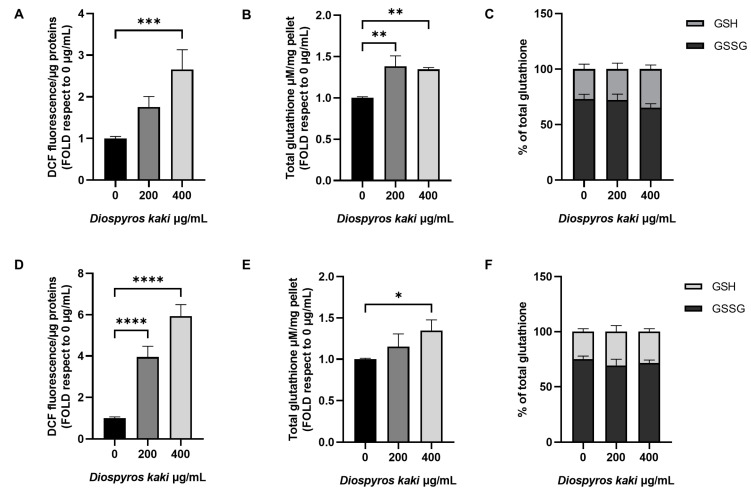
Analysis of reactive oxygen species following incubation with 5 µM H_2_DCFDA on E705 (**A**) and SW480 (**D**) cell lines untreated and treated with 200 and 400 µg/mL of *D. kaki* extract. Fluorescence is indicated as fold compared to the untreated control. Total glutathione level of the E705 (**B**) and SW480 (**E**) cell lines is expressed as fold compared to the untreated condition. GSSG and GSH contents expressed as a percentage of total glutathione are presented in panel (**C**,**F**) for the E705 and SW480 cell lines, respectively. Average values ± SE from three biological replicates are shown and three technical replicates are included in each biological replicate. Statistical significance: * *p* < 0.05, ** *p* < 0.01, *** *p* < 0.001, **** *p* < 0.0001.

Evaluation of oxidative stress markers showed a dose-dependent increase in cytosolic reactive oxygen species in both E705 and SW480 cells treated with *D. kaki* extract, as shown in Figure 4A,D. Total glutathione was also found to increase in both cell lines, although to a minor extent compared to the increase in ROS (Figure 4B,E). In particular, in E705 cells a 50% increase in total glutathione was detected after 200 µg/mL *D. kaki* extract administration, while no further increase was induced by the administration of 400 µg/mL extract; this suggests that the high ROS increase may not be adequately matched by molecular defense against reactive oxygen species. In SW480 cells, the increase in total glutathione was found to be dose-dependent, but at the maximum extract dose, it was only about 30% of the initial level, suggesting that it may be insufficient to balance increased oxidative stress. The ratio between reduced (GSH) and oxidized (GSSG) glutathione was found to be unaffected by extract administration. This unbalanced redox homeostasis, especially at the higher dose investigated, is likely responsible for apoptotic cell death probably by causing both cell and nuclear membrane damage and ruptures, in accordance with previous data on CRC and liver cancer cells [10].

### 3.4. Mitochondria Dysfunction Induced by D. kaki Extract Is Not Rescued by Glycolysis Upregulation

**Figure 5:**
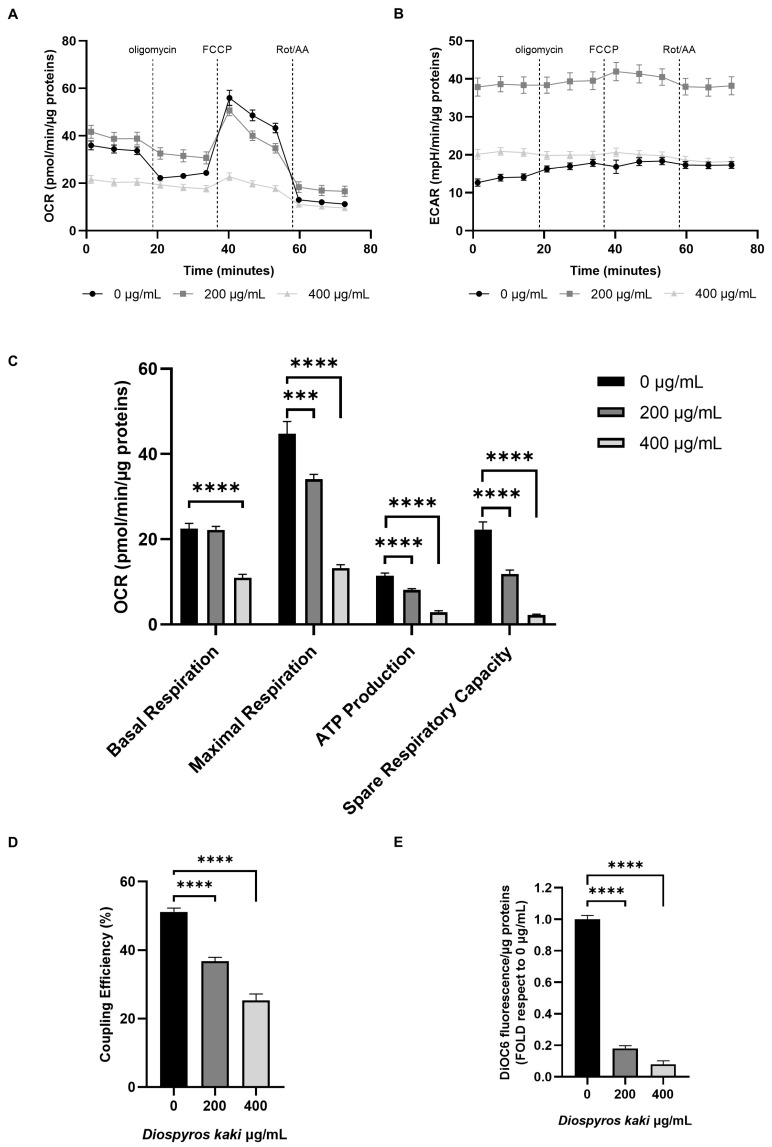
OCR and ECAR profiles following Cell Mito Stress Test on the E705 cell line untreated and treated for 24 h with 200 and 400 µg/mL D. kaki extract (**A**,**B**). Dotted lines indicate the time of addition of 1 µM oligomycin, 2 µM FCCP and 1 µM rotenone and antimycin A. Basal respiration, maximal respiration, ATP production, spare respiratory capacity (**C**), and coupling efficiency (**D**) are reported as mean ± SE of three biological replicates, each carried out in technical quadruplicate. Analysis of mitochondrial Δψ after incubation with 40 nm DiOC6 (**E**). Fluorescence is represented as fold compared to the untreated condition. Average values ± SE from three biological replicates are shown and three technical replicates are included in each biological replicate. Statistical significance: *** *p* < 0.001, **** *p* < 0.0001.

**Figure 6:**
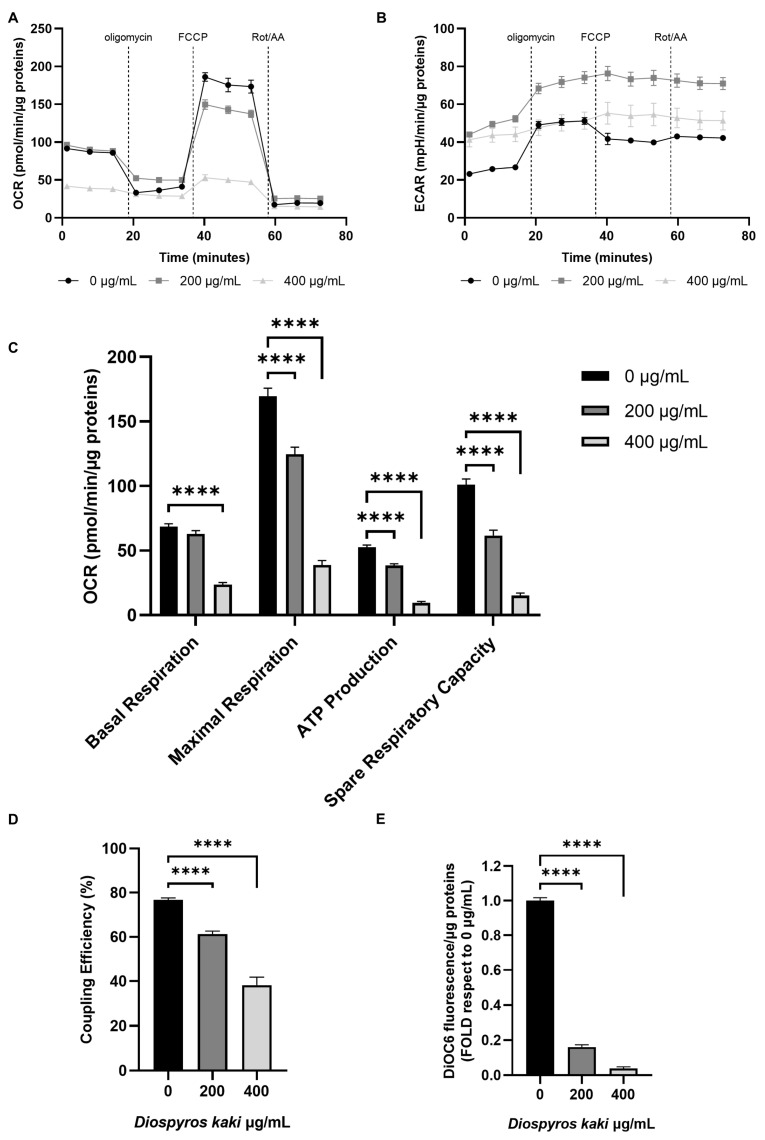
OCR and ECAR profiles following Cell Mito Stress Test on the SW480 cell line untreated and treated for 24 h with 200 and 400 µg/mL *D. kaki* extract (**A**,**B**). Dotted lines indicate the time of addition of 1 µM oligomycin, 2 µM FCCP and 1 µM rotenone and antimycin A. Basal respiration, maximal respiration, ATP production, spare respiratory capacity (**C**) and coupling efficiency (**D**) are reported as mean ± SE of three biological replicate, each carried out in technical quadruplicate. Analysis of mitochondrial Δψ after incubation with 40 nm DiOC6 (**E**). Fluorescence is represented as fold compared to the untreated condition. Average values ± SE from three biological replicates are shown and three technical replicates are included in each biological replicate. Statistical significance: **** *p* < 0.0001.

**Figure 7:**
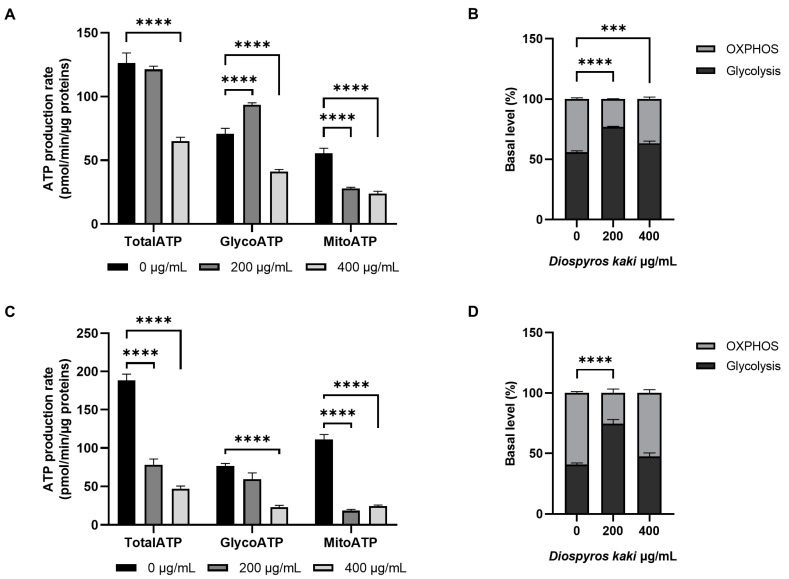
ATP Rate Assay. Total, glycolytic and mitochondrial ATP production rate in E705 (**A**) and SW480 (**C**) cell lines untreated and treated for 24 h with 200 and 400 µg/mL of *D. kaki* extract. The ratio between glycolytic and mitochondrial ATP production in E705 (**B**) and SW480 (**D**) cell lines is also reported. Average values ± SE from three biological replicates are shown and four technical replicates are included in each biological replicate. Statistical significance: *** *p* < 0.001, **** *p* < 0.0001.

The increase in oxidative stress detected following incubation with *D. kaki* extract prompted us to investigate mitochondrial functionality through Seahorse technology. Results, reported in Figure 5, Figure 6 and Figure 7, showed that *D. kaki* extract administration triggered mitochondrial dysfunction in both E705 and SW480 cells. Although SW480 cells showed a higher basal respiration rate, extract addition led in both cases to a dose-dependent decrease in both the maximal respiratory rate and the spare respiratory capacity (Figure 5A–C and Figure 6A–C). In particular, following incubation with 400 µg/mL extract, basal respiration was decreased to about 50 pmol/min/µg protein in SW480 cells and to 20 pmol/min/µg protein in E705 cells, showing only a minimal increase upon FCCP administration (Figure 5A and Figure 6A). This suggests that mitochondria are largely uncoupled, as shown in Figure 5D and Figure 6D. Accordingly, mitochondrial electrochemical potential decreased in both cell lines in a dose-dependent fashion (Figure 5E and Figure 6E). The loss of mitochondrial potential, being an indicator of mitochondrial damage, further supports our hypothesis of mitochondria-driven apoptosis [11].

ECAR evaluation, a pH measurement which is contributed to by both glycolysis and oxidative phosphorylation, showed a different pattern in the two different cell lines. In E705 cells (Figure 5B), the addition of 200 µg/mL *D. kaki* extract led to a marked ECAR increase, which is entirely due to glycolysis hyperactivation, as demonstrated by the fact that it was not lowered by electron transport inhibition through rotenone and antimycin A addition. Moreover, no ECAR variation was observed when ATP synthase was inhibited, through oligomycin addition, nor upon mitochondrial uncoupling through FCCP addition, showing that the basal ECAR increase was entirely due to glycolysis upregulation. When 400 µg/mL extract was added, basal ECAR increased to half the level reached upon addition of 200 µg/mL extract; in this case, the increase can also be attributed exclusively to glycolysis upregulation, as demonstrated by the fact that it remained unchanged after rotenone and antimycin administration.

In SW480 cells, the addition of either 200 or 400 µg/mL extract increased ECAR by two-fold. However, following treatment with 200 µg/mL extract, glycolysis could be further upregulated upon oligomycin addition, suggesting that it could rescue aerobic ATP production impairment. On the other hand, after treatment with 400 µg/mL extract, the glycolytic rate did not increase, following aerobic ATP synthesis inhibition.

Cancer cells normally thrive by upregulating glycolysis via the so-called Warburg effect, which leads to many metabolic rearrangements aimed at building a metabolic platform fit to support high-rate proliferation. Because of upregulated glycolysis, cells exhibiting the Warburg effect produce most of their ATP in an anaerobic way; however, following treatment with 400 µg/mL D. kaki extract, both cell lines cannot increase glycolytic rate by switching to the Warburg effect. This inability to switch towards the Warburg effect could be caused by the flavonoids’ capacity to impair aerobic glycolysis by inhibiting key glycolytic enzymes in cancer cells [31,32,33].

These data are in accordance with the relative contribution of glycolysis and oxidative phosphorylation to ATP synthesis reported in Figure 7. In E705 cells, total ATP was found to decrease only upon addition of 400 µg/mL D. kaki extract, in accordance with the high glycolytic basal level of this cell line (Figure 7A). The addition of 200 µg/mL extract led to an increase in glycolytic ATP production, due to glycolysis hyperactivation, and to a decrease in mitochondrial ATP production; however, following 400 µg/mL extract administration, both glycolytic and mitochondrial ATP production markedly decreased (Figure 7A). Therefore, E705 cells’ metabolism was shifted towards a more glycolytic phenotype by the addition of 200 µg/mL extract; on the other hand, it reverted to the original ratio between mitochondrial and glycolytic ATP production following treatment with 400 µg/mL extract, although in this case, total ATP production was strongly impaired (Figure 7B).

In SW480 cells, the addition of *D. kaki* extracts at either concentration led to a marked decrease in total ATP (Figure 7C). Both glycolytic and mitochondrial ATP production were impaired by the extract administration, in a dose-dependent way. However, following treatment with 200 µg/mL extract, glycolysis upregulation could limit glycolytic ATP decrease, while mitochondrial ATP production was already strongly impaired, switching SW480 cells’ metabolism towards a glycolytic phenotype (Figure 7D). After treatment with 400 µg/mL extract, both glycolytic and mitochondrial ATP production were seriously damaged, leading most cells to apoptotic death.

## 4. Conclusions

This work shows that *D. kaki* alcoholic extract is endowed with anticancer activity towards both E705 and SW480 cell lines. At the molecular level, *D. kaki* extract increases oxidative stress, which in turn leads to mitochondrial dysfunction. This causes a dramatic drop in ATP synthesis, which triggers apoptosis. Moreover, the ability of *D. kaki* extract to prevent cell switching to the Warburg effect, although still to be elucidated at molecular level, makes this extract extremely promising for CRC therapy. Although the concentrations used in this work are high, further studies will enable us to identify the compound(s) responsible for this proapoptotic effect, allowing lower concentrations to be used.

Besides implementing current research on phytochemicals, this work holds significant interest for society, especially in light of the 2030 objective “health and wellbeing”: the proapoptotic effect of *D. kaki* extract, which is highly specific towards cancer cells, paves the way for its use in both CRC therapy and prevention.

## Figures and Tables

**Table 1:** UHPLC-(―)-HRMS data of compounds detected in *D. kaki* leaf extract.

N	Compound	Molecular Formula	Rt (min)	[M-H]^−^ (*m*/*z*)	Error (ppm)	Diagnostic Product Ions (*m*/*z*)	MSI Level ^a^
1	(epi)Gallocatechin-(epi)gallocatechin	C_30_H_26_O_14_	2.2	609.1248	−0.2	483.0952, 441.0816, 423.0715, 305.0663, 177.0183, 125.0231	2
2	Gallocatechin	C_15_H_14_O_7_	4.5	305.0663	−1.0	167.0339, 165.0182, 137.0232, 125.0231	1
3	(epi)Gallocatechin-(epi)catechin	C_30_H_26_O_13_	4.7	593.1296	−0.7	467.0996, 441.0825, 425.0873, 407.0765, 303.0512, 289.0715	2
4	(epi)Catechin-(epi)gallocatechin	C_30_H_26_O_13_	5.0	593.1294	−1.2	467.0985, 441.0829, 423.0714, 305.0663, 289.0714, 287.0559	2
5	Hydroxyroseoside	C_19_H_30_O_9_	5.3	447.1867 ^b^	−1.3	371.1716, 239.1287	3
6	Procyanidin B1	C_30_H_26_O_12_	5.6	577.1342	−1.2	451.1023, 425.0874, 407.0766, 299.0557, 289.0715, 287.0557	1
7	Catechin	C_15_H_14_O_6_	5.9	289.0713	−1.6	151.0389, 149.0233, 137.0232, 125.0231	1
8	Coumaroyl-hexoside-pentoside	C_20_H_26_O_12_	6.1	457.1347	−1.0	325.0926, 1630.390, 119.0490	2
9	Roseoside	C_19_H_30_O_8_	6.3	431.1914 ^b^	−2.0	223.1321, 205.0498	2
10	Roseoside pentoside	C_24_H_38_O_12_	6.4	563.2335 ^b^	−2.1		3
11	Iridoid glycoside	C_19_H_32_O_8_	7.0	433.2077 ^b^	−0.3		4
12	Iridoid glycoside	C_19_H_28_O_10_	7.2	415.1606	−0.9		4
13	Myricetin 3-O-hexoside 1	C_21_H_20_O_13_	8.5	479.0829	−0.3	317.0283, 316.0220, 287.0195, 271.0246, 178.9976, 151.0026	2
14	Myricetin 3-O-hexoside 2	C_21_H_20_O_13_	8.8	479.0829	−0.3	317.0280, 316.0220, 287.0196, 271.0246, 178.9975, 151.0026	2
15	Quercetin-3-O-hexoside-deoxyhexoside	C_27_H_30_O_16_	10.2	609.1464	0.7	301.0318, 300.0271, 271.0246, 255.0296, 178.9969, 151.0022	2
16	Iridoid glycoside 1	C_24_H_42_O_11_	10.5	551.2708 ^b^	−0.2		4
17	Iridoid glycoside 2	C_24_H_42_O_11_	10.7	551.2705 ^b^	−0.6		4
18	Quercetin-3-O-hexoside	C_21_H_20_O_12_	11.3	463.0875	−1.3	301.0343, 300.0272, 271.0245, 255.0295, 178.9974, 151.0025	2
19	Iridoid glycoside 3	C_24_H_42_O_11_	11.4	551.2700 ^b^	−1.8		2
20	Quercetin-3-O-glucoside (isoquercitrin)	C_21_H_20_O_12_	11.6	463.0874	−1.7	301.0342, 300.0272, 271.0245, 255.0294, 178.9973, 151.0026	1
21	Kaempferol-3-O-hexoside-deoxyhexoside	C_27_H_30_O_15_	11.9	593.1506	−0.8	285.0378, 284.0322, 255.0294, 227.0343, 151.0022	2
22	Laricitrin 3-O-hexoside	C_22_H_22_O_13_	12.1	493.0985	−0.5	331.0461, 330.0375, 316.01930, 315.0144, 287.0195, 178.9975, 151.0022	2
23	Quercetin-7(4′)-O-galloylhexoside 1	C_28_H_24_O_16_	12.4	615.0981	−1.6	313.0559, 301.0349, 178.9975, 169.0129, 151.0025	2
24	Quercetin-7(4′)-O-galloylhexoside 2	C_28_H_24_O_16_	12.7	615.0983	−1.6	463.0864, 313.0566, 301.0351, 178.9977, 169.0127, 151.0026	2
25	Kaempferol-3-O-hexoside	C_21_H_20_O_11_	12.6	447.0924	−2.1	285.0392, 284.0324, 255.0296, 227.0344, 151.0025	2
26	Kaempferol-3-O-glucoside (Astragalin)	C_21_H_20_O_11_	13.3	447.0923	−2.2	285.0394, 284.0323, 255.0295, 227.0343, 151.0026	1
27	Kaempferol-3-O-pentose	C_20_H_18_O_10_	13.9	417.0825	−0.4	285.0387, 284.0323, 255.0295, 227.0343, 151.0023	2
28	Kaempferol 7(4′)-O-galloylhexoside 1	C_28_H_24_O_15_	14.2	599.1032	−1.9	313.0563, 285.0402, 257.0455, 229.0498, 169.0132, 151.0025	2
29	Kaempferol 7(4′)- O-galloylhexoside 2	C_28_H_24_O_15_	14.6	599.1033	−1.7	313.0562, 285.0402, 257.0452, 229.0502, 169.0133, 151.0025	2
30	Kaempferol 7(4′)-O-galloylpentoside 1	C_27_H_22_O_14_	15.8	569.0937	0.2	285.0402, 283.0457, 257.0452, 229.0500, 169.0130, 151.0025	2
31	Kaempferol 7(4′)-O-galloylpentoside 2	C_27_H_22_O_14_	16.0	569.0939	0.5	285.0402, 283.0458, 257.0453, 229.0500, 169.0129, 151.0026	2

^a^ according to metabolomics standards initiative (MSI); ^b^ corresponding to formic acid adduct [M+FA-H]^−^.

## Data Availability

The original contributions presented in the study are included in the article, further inquiries can be directed to the corresponding authors.
